# Rationale and design of a phase II trial of pyrotinib in combination with nab-paclitaxel as adjuvant therapy for N0/N1mi, HER2 + early breast cancer (PHAEDRA)

**DOI:** 10.1186/s12885-022-09346-1

**Published:** 2022-03-14

**Authors:** Changjun Wang, Yidong Zhou, Yan Lin, Feng Mao, Jinghong Guan, Xiaohui Zhang, Songjie Shen, Xuejing Wang, Yanna Zhang, Bo Pan, Ying Zhong, Li Peng, Xi Cao, Ru Yao, Xingtong Zhou, Chi Xu, Ying Xu, Qiang Sun

**Affiliations:** Department of Breast Surgery, Peking Union Medical College Hospital, No.41 DaMuCang Lane, Xicheng District, Beijing, 100032 China

**Keywords:** Pyrotinib, Nab-paclitaxel, Adjuvant therapy, Human epidermal growth factor receptor 2, Breast cancer, Protocol

## Abstract

**Background:**

The de-escalation treatment in patients with low-risk HER2-positive early breast cancer (eBC) is an attractive strategy to avoid unnecessary treatment and improve the quality of life of patients. Pyrotinib, a novel irreversible pan-HER2 tyrosine kinase inhibitor (TKI), has shown efficacy in patients with advanced HER2-positive breast cancer. Meanwhile, nanoparticle albumin-bound (nab)-paclitaxel reveals survival benefit over solvent-based paclitaxel and eliminates the toxicities associated with the solvent. However, the efficacy and safety of pyrotinib in combination with nab-paclitaxel as adjuvant therapy for low-risk HER2 + eBC patients have not been evaluated.

**Methods:**

This is a multicenter, open-label, single-arm phase II study. A sample size of 261 patients with tumor ≤ 3 cm, lymph node-negative (N0) or micrometastatic (N1mi), HER2 + breast cancer will be recruited. Eligible patients will receive nab-paclitaxel 260 mg/m^2^ once every 3 weeks for 12 weeks and pyrotinib 400 mg once daily for one year. The primary endpoint is invasive disease-free survival. A sub-study will be conducted to investigate different prophylactic strategies for diarrhea, which is the most common adverse event of pan-HER TKIs. One hundred and twenty patients from the main study will be randomly (1:1) allocated to receive loperamide either during the first cycle (4 mg tid on days 1–7, then 4 mg bid on days 8–21) or the first 2 cycles (4 mg tid on days 1–7, then 4 mg bid on days 8–42). The primary endpoint of the sub-study is the incidence of grade ≥ 3 diarrhea.

**Discussion:**

This is the first prospective study of pyrotinib in combination with nab-paclitaxel as adjuvant therapy for patients with low-risk HER2-positive eBC. It would probably provide robust evidence for de-escalating strategy of HER2-positive eBC and appropriate management for pyrotinib-related diarrhea.

**Trial registration:**

ClinicalTrials.gov Identifier: NCT04659499. Registered on December 9, 2020.

## Background

For breast cancer, human epidermal growth factor receptor 2 (HER2)/ErbB-2 gene amplification or protein overexpression is associated with poor survival [1, 2]. The invention of the first anti-HER2 antibody trastuzumab significantly improved the survival of patients with HER2 + breast cancer. It has been widely used for breast cancer treatment in (neo)adjuvant and metastatic settings. Afterwards, with the emergence of novel anti-HER2 antibodies, antibody drug conjugates and tyrosine kinase inhibitors (TKIs), dual HER2-targeted therapy became the mainstay for high-risk HER2 + breast cancer (e.g. APHINITY and ExteNET trials) [3, 4]. However, the optimal adjuvant regimen for low-risk HER2 + breast cancer remains inconclusive.

Previous randomized controlled trials (RCTs) established trastuzumab-based therapy as standard of care for patients with HER2 + , early breast cancer [5–7], but only a small proportion (0 to < 33%) of low-risk patients were included. Jones et al*.* explored the adjuvant therapy with four cycles of docetaxel and cyclophosphamide in combination with one year of trastuzumab in early breast cancer. The 2-year disease-free survival (DFS) and overall survival rates were 97.8% and 99.2%, respectively [8]. The APT trial firstly reported data of weekly paclitaxel plus trastuzumab as adjuvant therapy in patients with tumor (T) ≤ 3 cm, lymph node-negative (N0), HER2 + breast cancer. The 3-year invasive DFS (iDFS) rate was 98.7% [9]. Although mono anti-HER2 antibody combined with single-agent chemotherapy has become a recommended adjuvant regimen for node-negative, small HER2-positive tumors, there is scarcity of evidence on TKIs for HER2 + low-risk breast cancer.

Pyrotinib, a novel irreversible pan-ErbB receptor TKI targeting epidermal growth factor receptor (EGFR), HER2 and HER4, showed promising efficacy for metastatic breast cancer. A phase II trial demonstrated that pyrotinib plus capecitabine significantly increased median progression-free survival (PFS) compared with lapatinib plus capecitabine (18.1 vs. 7.0 months; hazard ratio [HR], 0.363; 95% confidence interval [CI]: 0.228–0.579; *P* < 0.001) in patients with HER2 + metastatic breast cancer [10]. The safety profile also proved that this combination therapy was well-tolerated [10]. Another phase III PHOEBE trial compared pyrotinib plus capecitabine with lapatinib plus capecitabine for HER2 + local relapsed or metastatic breast cancer. The interim analysis showed that the median PFS was significantly improved with pyrotinib plus capecitabine (12.5 months; 95% CI: 9.7-not reached) compared with 6.8 months (95%CI: 5.4–8.1) in the lapatinib plus capecitabine group (HR, 0.39; 95%CI: 0.27–0.56; *P* < 0.001) [11]. Some phase 2 studies have also investigated the role of pyrotinib for patients with HER2 early or locally advanced breast cancer in the neoadjuvant setting [12–14].

Regarding the chemotherapy backbone, nanoparticle albumin-bound (nab)-paclitaxel exhibited its therapeutic advantage over solvent-based paclitaxel and was widely used in combination with immunotherapy, such as programmed death-ligand 1 antibody [15]. Nab-paclitaxel was developed with the preservation of the antitumor activity of paclitaxel, and meanwhile decreased solvent-related toxicities. Consequently, the dosage of nab-paclitaxel could increase with acceptable incidence of adverse events (AEs) and result in improved efficacy [16, 17]. Study by Gradishar et al*.* led to the approval of nab-paclitaxel and demonstrated that nab-paclitaxel at 260 mg/m^2^ every 3 weeks achieved a higher overall response rate (33% vs. 19%; *P* = 0.001) and a longer time to progression (23.0 vs. 16.9 weeks; HR, 0.75; *P* = 0.006) compared with solvent-based paclitaxel at 175 mg/m^2^ every 3 weeks in patients with previously untreated, metastatic breast cancer [16]. Grade 4 neutropenia was less frequent among patients with nab-paclitaxel compared with those with solvent-based paclitaxel (9% vs. 22%; *P* < 0.001) [16]. The neoadjuvant phase III GeparSepto trial revealed that the pathological complete response rate was higher with nab-paclitaxel (233 [38.4%] patients) than with solvent-based paclitaxel (174 [29.0%] patients) [17]. Patients treated with nab-paclitaxel also had a significantly better 4-year iDFS rate compared with solvent-based paclitaxel (84.0% vs. 76.3%; HR, 0.66; 95%CI: 0.51–0.86; *P* = 0.002) [17].

Therefore, the main goal for the present study is to assess the efficacy and safety of pyrotinib in combination with nab-paclitaxel as adjuvant therapy for patients with low-risk HER2 + breast cancer.

Moreover, the second goal for the present study is to focus on the management of TKI-related diarrhea. Diarrhea is the most common AE of pan-HER TKIs. Grade ≥ 3 diarrhea occurred in 30.6% of patients treated with pyrotinib plus capecitabine in the PHOEBE trial [11]. In the ExteNET study of neratinib (another pan-HER TKI), which no mandated antidiarrheal prophylaxis was administered, grade 3–4 diarrhea was observed in 40% of patients and 17% discontinued neratinib due to diarrhea [18]. A phase II CONTROL study investigated several strategies to improve tolerability of neratinib. Loperamide could effectively reduce the incidence of grade 3 diarrhea (31%) which was lower than the ExteNET study and no grade 4 diarrhea was observed [19]. It could probably serve as a useful adjunct for pyrotinib. Given the unknown incidence and severity of diarrhea with pyrotinib plus nab-paclitaxel and no consensus on therapeutic or prophylactic strategy of pyrotinib-related diarrhea, a sub-study will be conducted to investigate the effect of different prophylactic strategies with loperamide for diarrhea.

## Methods/design

### Study design and treatment

This multicenter, open-label, single-arm phase II trial (ClinicalTrials.gov Identifier: NCT04659499) is conducted in accordance with the Declaration of Helsinki and Good Clinical Practice. The protocol has been approved by the Ethics Committee of Peking Union Medical College Hospital on October 27, 2020 (approval number: HS-2617).

**Fig. 1:**
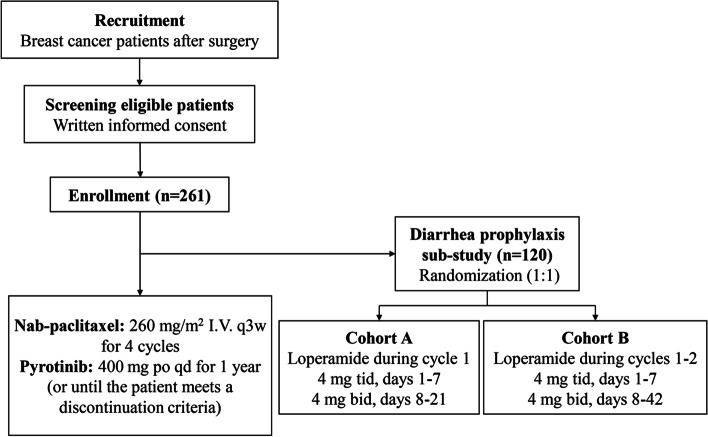
Study design

Within 90 days after appropriate surgical resection with sentinel lymph node biopsy or axillary lymph node dissection, patients will receive intravenous administration of nab-paclitaxel 260 mg/m^2^ once every 3 weeks for 12 weeks (i.e. 4 cycles) and oral pyrotinib 400 mg once daily for 1 year (Fig. 1). The treatment will be discontinued for disease recurrence, metastasis, intolerable toxicity, withdrawal of consent, or other reason judged by the investigator. Postoperative radiotherapy is decided by the investigator. Adjuvant hormonal therapy is recommended for patients with hormone receptor-positive disease after the completion of nab-paclitaxel therapy.

### Inclusion criteria

Patients are eligible if they are women aged 18–75 years; have histologically confirmed diagnosis of HER2 + primary invasive breast cancer with an immunohistochemical staining intensity of 3 + , or 2 + with HER2 gene amplification detected by in situ hybridization (ratio of HER2 to chromosome 17 centromere ≥ 2.0); have T ≤ 3 cm disease with pathologically confirmed N0 or one lymph node micrometastasis (N1mi); have known hormone receptor status; have Eastern Cooperative Oncology Group performance status 0–1; have adequate organ function (neutrophil count ≥ 1.5 × 10^9^/L; platelet count ≥ 90 × 10^9^/L; hemoglobin ≥ 90 g/L; total bilirubin ≤ 1.5 × upper limit of normal [ULN]; alanine transaminase and aspartate transaminase ≤ 3 × ULN; blood urea nitrogen and serum creatinine ≤ 1.5 × ULN and creatinine clearance rate ≥ 50 mL/min [Cockcroft-Gault formula]; left ventricular ejection fraction ≥ 50%; Fridericia-corrected QT interval ≤ 480 ms); and sign the informed consent.

### Exclusion criteria

The exclusion criteria were: clinical or radiologic evidence of local or regional disease recurrence or metastasis before the enrollment; previous treatment with chemotherapeutic drugs, or tyrosine kinase inhibitors targeting HER2 (such as lapatinib, neratinib and pyrotinib); other malignancies within 5 years, except for cured cervical carcinoma in situ, cutaneous basal cell carcinoma, and cutaneous squamous cell carcinoma; inability to swallow, chronic diarrhea, or intestinal obstruction; allergy to the components of study drug; history of immunodeficiency disease, including acquired immune deficiency syndrome, viral hepatitis C, active viral hepatitis B and other immunodeficiency diseases; history of organ transplantation; pregnant or lactating women, or women who are unwilling to take effective contraception during the study period; any heart disease, including arrhythmia requiring medical therapy or of clinical significance, myocardial infarction, heart failure and other heart diseases; concomitant diseases seriously affecting the patient safety or the completion of study, such as uncontrolled hypertension, severe diabetes mellitus and active infection; history of definite neurological or psychiatric disorders; concomitant use of CYP3A4 inhibitors or inducers, or QT-prolonging drugs; or participating in other clinical trials within 4 weeks.

### Endpoints

The primary endpoint is iDFS, defined as time from the initiation of pyrotinib plus nab-paclitaxel to ipsilateral invasive breast cancer recurrence, regional invasive breast cancer recurrence, distant recurrence, contralateral invasive breast cancer, or any-cause death. The secondary endpoints are safety and tolerability.

### Biomarkers

The blood samples, stool samples and tumor tissues (when available) will be collected for exploratory pharmacodynamics and predictive biomarkers analysis. Next-generation sequencing of genes, such as *PIK3CA*, *TP53*, *PTEN*, and *AKT*, will be performed on circulating tumor DNA and genomic DNA of blood and tumor samples. Metagenomic sequencing will be employed to study gut microbiota using stool samples.

### Sample size calculation

The group-sequential Poisson test based on the number of events of invasive disease or any-cause death was used for sample size calculation. In designing the trial, a 3-year event rate of ≥ 12.4% was considered to be unacceptable in this population, and ≤ 7.2% would be considered as a success [20, 21]. Assuming that the one-sided significance level is 5% and the power is 80%, the sample size should be 253. Considering a dropout rate of 10%, the target number of patients was determined as 261.

### Statistical analysis

Efficacy will be analyzed in the full analysis set (FAS) and per-protocol set (PPS), and FAS is the main analysis set. FAS includes all enrolled patients with at least one dose of study drug. PPS includes patients who received at least one dose of study drug without major protocol deviation. Safety will be analyzed in the safety set, which includes all patients with at least one dose of study drug and at least one safety assessment.

iDFS will be plotted using Kaplan–Meier method for the efficacy analysis. AEs and serious AEs will be summarized for the safety analysis.

### Sub-study design

A sub-study will be conducted to investigate the effect of different prevention strategies of diarrhea. A total of 120 patients from the main study who sign the sub-study informed consent will enter the sub-study. Patients will be randomly (1:1) assigned to two cohorts. Cohort A will receive loperamide during cycle 1 of adjuvant therapy with pyrotinib plus nab-paclitaxel, at a dose of 4 mg three times a day on days 1–7 and 4 mg twice a day on days 8–21. Cohort B will receive loperamide during cycles 1 and 2 of adjuvant therapy, at a dose of 4 mg three times a day on days 1–7 and 4 mg twice a day on days 8–42.

When grade 1–2 diarrhea with complications (including but not limited to abdominal cramping, grade ≥ 2 nausea/vomiting, decreased Eastern Cooperative Oncology Group performance status, fever, pyemia, neutropenia, bleeding, and dehydration) or grade 3 diarrhea occurs, pyrotinib treatment will be interrupted and resumed until recovery to grade 0–1 without complications. Dose reduction is not required after recovery at the first time, but should be reduced to 320 mg at next time and to 240 mg at most. If the continuous interruption time is more than 28 days, patients will be withdrawn from the study. When grade 4 diarrhea occurs, pyrotinib will be discontinued permanently.

The primary endpoint of the sub-study is the incidence of grade ≥ 3 diarrhea from the first dose to 28 days after the last dose of the study drug. The secondary endpoints of the sub-study include the incidence and severity of diarrhea during the first 2 cycles of adjuvant therapy, the incidence and severity of constipation, the onset time, frequency and duration of grade ≥ 3 diarrhea, relationship between diarrhea and study drugs, the incidence of dose reduction, discontinuation and hospitalization due to diarrhea, and quality of life questionnaire score using the Functional Assessment of Cancer Therapy-Breast.

## Discussion

In most guidelines, the recommended adjuvant regimen for HER2 + early breast cancer patients is based on the treatment containing trastuzumab and taxanes [22, 23]. Although four pivotal RCTs (HERA, BCIRG-006, NSABP B-31 and NCCTG N9831) established the standard of care of trastuzumab [5–7], efficacy data are scarce in small-tumor, N0/N1mi, HER2 + low-risk breast cancer population.

Low-risk patients with small-tumor, N0/N1mi breast cancer generally had a better prognosis and received less benefit from adjuvant therapy. The subgroup analysis of BCIRG-006 trial demonstrated a high 5-year DFS rate (84%) with adjuvant AC-T regimen (doxorubicin and cyclophosphamide followed by docetaxel) in patients with small (< 2 cm) HER2 + breast cancer, but the addition of trastuzumab (AC-TH) only increased 5-year DFS rate by 3% [6]. The adjuvant TCH regimen (docetaxel and carboplatin plus trastuzumab) showed comparable 5-year DFS benefit to the AC-TH (85% vs. 87%) and AC-T regimens (85% vs. 84%) [6]. Thus, de-escalation strategy with less toxic chemotherapy and HER2-targeted agents received much attention. The adjuvant APT trial adopted weekly paclitaxel plus trastusumab and achieved a 3-year iDFS rate up to 98.7% in patients with T ≤ 3 cm, N0/N1mi, HER2 + breast cancer [9]. Similarly, the study by Jones et al*.* proved that adjuvant docetaxel and cyclophosphamide plus trastuzumab could reach a 3-year iDFS of 96.9% in patients with HER2-amplified early breast cancer [8]. Given the extremely great survival benefit in the abovementioned de-escalation studies, less intensive chemotherapy with mono HER2-targeted therapy can be a promising combination that bring better safety profile without compromising the efficacy. Small-molecule anti-HER2 TKI could be a reasonable option as part of this novel combination. However, the evidence on TKIs in this low-risk population is scarce.

Pyrotinib as a novel irreversible pan-ErbB receptor TKI targets not only HER2, but also EGFR and HER4. Theoretically, it has more potent anti-HER2 effect. Previous studies demonstrated that pyrotinib had a better antitumor activity over lapatinib when combined with capecitabine for HER2 + metastatic breast cancer, leading to the approval of pyrotinib in China [10, 11]. In the interim analysis of phase III PHOEBE trial, 67.2% of patients responded to pyrotinib plus capecitabine, with a median duration of response of 11.1 months (95%CI: 9.7-not reached) [11]. This durable effect of pyrotinib plus capecitabine was also observed in trastuzumab-resistant patients, with a nearly doubled PFS benefit compared with lapatinib plus capecitabine (12.5 vs. 6.9 months; HR, 0.60; 95%CI: 0.29–1.21) [11]. For the chemotherapy component of this combination, nab-paclitaxel can be an appropriate option. First, it exhibited therapeutic and safety advantages over solvent-based paclitaxel in early and advanced breast cancer [16, 17]. Study by Gradishar et al*.* demonstrated higher overall response rate (33% vs. 19%; *P* = 0.001), longer time to progression (23.0 vs. 16.9 weeks; HR, 0.75; *P* = 0.006) and lower incidence of grade 4 neutropenia (9% vs. 22%; *P* < 0.001) with nab-paclitaxel versus solvent-based paclitaxel in patients with previously untreated, metastatic breast cancer, despite a 49% higher paclitaxel dose [16]. The use of nab-paclitaxel also led to higher pathological complete response rate (38.4% vs. 29.0%; *P* = 0.001) and better 4-year iDFS rate (84.0% vs. 76.3%; HR, 0.66; 95%CI: 0.51–0.86; *P* = 0.002) compared with solvent-based paclitaxel in the neoadjuvant phase III GeparSepto trial [17]. Second, compared with other chemotherapy agent, such as capecitabine, nab-paclitaxel leads to lower incidence of diarrhea. There is no great overlap of AEs between nab-paclitaxel and pyrotinib. Finally, nab-paclitaxel has been widely used in immunotherapy trials because it does not need glucocorticoid premedication. This could provide opportunity to integrate immunotherapy with the current strategy for future study.

Taken together, the present study will be the first one to assess the efficacy and safety of pyrotinib plus nab-paclitaxel as adjuvant therapy in patients with T ≤ 3 cm, N0/N1mi, HER2 + breast cancer. Small-molecule oral TKI combined with low-toxicity chemotherapeutic drug will probably provide a novel regimen for low-risk HER2 + breast cancer with less AE, comparable efficacy, better compliance, and reduced socio-economic burden compared with current standard adjuvant therapy.

The second goal of the present study would focus on the management of TKI-related diarrhea. Severe diarrhea may lead to unnecessary dose reduction or discontinuation and undermine the efficacy and quality of life. According to available data on pyrotinib (published and unpublished), the most frequent pyrotinib-related AE was diarrhea. A phase II trial showed that grade 3–4 diarrhea occurred in 10 (15.4%) of 65 patients in the pyrotinib plus capecitabine group [10]. In the phase III PHOEBE trial, the incidence of grade 3 diarrhea with pyrotinib plus capecitabine was 30.6% [11]. Diarrhea was considered to be a universal side-effect for anti-HER2 TKIs. For another pan-HER TKI, neratinib, the ExteNET study reported that 40% of patients had grade 3–4 diarrhea and 17% lead to treatment discontinuation [18]. Consequently, a phase II CONTROL study was designed to investigate the appropriate therapeutic and prophylactic strategy for management of TKI-related diarrhea. In CONTROL study, four cohorts with different antidiarrheal regimens prophylaxis and one cohort with neratinib dose escalation without any antidiarrheal agents for prophylaxis. All cohorts showed reduced rates of grade ≥ 3 diarrhea versus ExteNET (40%), and no grade 4 diarrhea occurred. The incidence of grade 3 diarrhea was 31%, 28%, 21%, 32%, and 15% in patients with loperamide alone, budesonide + mandatory loperamide, colestipol + mandatory loperamide, colestipol + as-needed loperamide, and neratinib dose escalation, respectively [19]. Most grade 3 diarrhea and treatment discontinuation due to diarrhea occurred at the first month. Loperamide-based antidiarrheal prophylaxis was effective but neratinib dose escalation seemed to be a more promising strategy as it eliminated mandatory prophylaxis and related side-effects. However, the CONTROL study did not reveal the relationship between different antidiarrheal strategies and efficacy. Whether the dose escalation regimen had an impact on the efficacy of neratinib adjuvant therapy is unclear. Based on these findings, a sub-study was designed to explore the efficacy of loperamide prophylaxis on pyrotinib-related diarrhea and the impact of loperamide dosage and duration (4 mg three times daily on days 1–7 in both cohorts, and 4 mg twice daily on days 8–21 in cohort A or on days 8–42 in cohort B).

The design of a randomized controlled sub-study in a large-scale single-arm trial is another highlight of the present trial. This study intends to answer two questions in the same study population and generate both high-level evidence in the hierarchy of evidence-based medicine. The underlying rationale is that the sample size for designing an RCT for sub-study is much less than that needed to evaluate survival for the main study. So, in this scenario, an RCT design for the present sub-study could be easily embedded into the whole study instead of conducting pre-specified or post-hoc subgroup analyses. It also has several other advantages: First, it provides more reliable evidence than pre-specified or post-hoc subgroup analyses. Second, it only needs 120 patients to be recruited, which is almost half of the whole study population. Third, the sub-study can be completed before the completion of the survival follow-up, and maybe even earlier than the end of recruitment period for the main study. Hence, it can timely deliver new guidance for the prophylaxis of AE. From the perspective of clinical trial methodology, this design has the same purpose as I-SPY2 trial in the neoadjuvant setting [24]. The I-SPY 2 trial was mentioned as a “paradigm shift” study with adaptive design to accelerate the development and delivery of more effective therapies [25]. It adopted an adaptive design and evaluated several study drugs simultaneously to improve efficiency. Similarly, our study provides a novel modality that takes the advantage of large sample size of adjuvant trials and evaluates different investigations and endpoints in a more efficient way.

## Data Availability

Not applicable.

## Abbreviations

AC-T: Doxorubicin and cyclophosphamide followed by docetaxel
AC-TH: Doxorubicin and cyclophosphamide followed by docetaxel and trastuzumab
AE: Adverse event; CI: confidence interval
DFS: Disease-free survival
EGFR: Epidermal growth factor receptor
FAS: Full analysis set
HER2: Human epidermal growth factor receptor 2
HR: Hazard ratio
iDFS: Invasive disease-free survival
NAB: Nanoparticle albumin-bound
N0: Lymph node-negative
N1mi: One lymph node micrometastasis
PFS: Progression-free survival
PPS: Per-protocol set
RCT: Randomized controlled trial
TCH: Docetaxel and carboplatin plus trastuzumab
TKI: Tyrosine kinase inhibitor
ULN: Upper limit of normal
